# Comparison of Treatment Zone Decentration Between VISUMAX 500 and VISUMAX 800 Keratorefractive Lenticule Extraction Using Anterior Segment Optical Coherence Tomography

**DOI:** 10.3390/jcm15207851

**Published:** 2026-10-12

**Authors:** Tae Keun Yoo, Si-Hoon Park, Sang-Mok Lee

**Affiliations:** 1 Hangil Eye Hospital, Incheon 21388, Republic of Korea; fawoo2@yonsei.ac.kr (T.K.Y.);; 2 Bright St. Mary Eye Clinic (BSM Eye Clinic), Seoul 06232, Republic of Korea; 3 Department of Ophthalmology, Catholic Kwandong University College of Medicine, Incheon 22711, Republic of Korea

**Keywords:** SMILE, decentration, anterior segment optical coherence tomography, VISUMAX

## Abstract

**Background/Objectives**: To compare treatment-zone decentration after small-incision lenticule extraction (SMILE) performed with VISUMAX 500 and VISUMAX 800 using corneal thickness difference maps derived from anterior segment optical coherence tomography. **Methods**: This retrospective comparative study included 44 eyes of 22 participants who underwent SMILE with VISUMAX 500 or VISUMAX 800 by a single surgeon and had preoperative and 3-month postoperative CASIA2 examinations. Treatment-zone centers were primarily identified manually relative to the corneal vertex. Between-platform differences were analyzed using generalized estimating equations (GEE) accounting for inter-eye correlation, with additional adjustment for preoperative cylinder. Participant-level analyses and semi-automated radial color-symmetry localization were performed as sensitivity analyses. **Results**: Twenty-two eyes of 11 participants were included in each group. Mean radial decentration was 0.219 ± 0.134 mm with VISUMAX 500 and 0.225 ± 0.171 mm with VISUMAX 800. The mean between-group difference was 0.006 mm (95% confidence interval [CI], −0.074 to 0.085; *p* = 0.890), and remained similar after adjustment for preoperative cylinder (0.006 mm; 95% CI, −0.082 to 0.094; *p* = 0.888). Participant-level analysis likewise showed no evidence of a between-group difference (mean difference, 0.0056 mm; 95% CI, −0.083 to 0.094; exact permutation *p* = 0.907). Reanalysis using semi-automated localization yielded a similarly small between-platform difference (0.0114 mm; 95% CI, −0.063 to 0.086; *p* = 0.763). **Conclusions**: In this small single-surgeon cohort, treatment-zone decentration did not differ significantly between VISUMAX 500 and VISUMAX 800. The findings were consistent across participant-level and semi-automated sensitivity analyses. Because the study was not designed as an equivalence or noninferiority trial, these results should be interpreted as an absence of evidence for a between-platform difference rather than evidence of equivalent centration performance.

## 1. Introduction

Small-incision lenticule extraction (SMILE) is a flapless corneal refractive procedure in which an intrastromal lenticule is created with a femtosecond laser and removed through a small incision [1]. Accurate centration of the lenticule is important because treatment-zone decentration may induce higher-order aberrations and adversely affect postoperative visual quality, particularly in eyes with astigmatism [2,3].

With VISUMAX 500 (Carl Zeiss Meditec AG, Jena, Germany), centration is largely dependent on manual alignment by the surgeon, and cyclotorsional compensation is limited. VISUMAX 800 (Carl Zeiss Meditec AG, Jena, Germany) was subsequently introduced with centration and cyclotorsion-assistance functions, including CentraLign and OcuLign, with the aim of improving treatment alignment and surgical reproducibility [4,5]. Although these technological advances may improve treatment alignment, direct comparative evidence regarding postoperative treatment-zone centration between VISUMAX 500 and VISUMAX 800 remains limited and may depend on the centration workflow and measurement method [6].

Anterior segment optical coherence tomography allows for objective assessment of corneal thickness changes after refractive surgery. In this study, we used preoperative and postoperative corneal thickness difference maps obtained with CASIA2 (Tomey Corporation, Nagoya, Japan) to compare treatment-zone decentration after SMILE performed with VISUMAX 500 and VISUMAX 800.

## 2. Materials and Methods

### 2.1. Study Design and Participants

This retrospective comparative study was conducted at Hangil Eye Hospital and included patients who underwent SMILE with either VISUMAX 500 or VISUMAX 800 between October 2023 and June 2024. All procedures in both groups were performed by the same experienced refractive surgeon (S.M.L.) to minimize variability related to surgical technique and centration.

Eyes were included if preoperative and 3-month postoperative anterior segment optical coherence tomography (AS-OCT) examinations using CASIA2 were available and of sufficient quality for generation and assessment of corneal thickness difference maps (CASIA2 differential pachymetry maps). Both eyes were included when eligible.

The study adhered to the tenets of the Declaration of Helsinki and was approved by the Institutional Review Board of Hangil Eye Hospital (approval No. Hangil IRB-25001). The requirement for written informed consent was waived because of the retrospective nature of the study.

The VISUMAX 800 procedures were performed during the same early implementation period as a previously reported institutional cohort of SMILE Pro cases [7]. The present analysis, however, was restricted to procedures performed by a single surgeon and to eyes with paired preoperative and postoperative AS-OCT examinations, with treatment-zone centration as the primary outcome. The VISUMAX 500 and VISUMAX 800 procedures were performed during substantially overlapping calendar periods rather than as strictly sequential cohorts. Platform selection was not randomized. Because the two platforms were offered under different pricing policies, patients received an explanation of the available surgical platforms and associated costs and selected the platform according to their preference. Because eligibility required paired preoperative and 3-month postoperative CASIA2 examinations, the analyzed cohort did not represent a consecutive chronological series of all procedures performed during the study period.

### 2.2. Surgical Technique and Centration

All procedures were performed under topical anesthesia using either the VISUMAX 500 or VISUMAX 800 femtosecond laser platform. The programmed optical zone diameter was 6.5 mm in all eyes in both the VISUMAX 500 and VISUMAX 800 groups.

For VISUMAX 500, no external diagnostic device was linked to the laser system for centration or cyclotorsional compensation. Reference marks were placed on the cornea with a marking pen before docking for cyclotorsional adjustment when the amount of astigmatism was higher than 2.00 D or at least half of the spherical power. During docking, patients were instructed to fixate on the internal green fixation light, and the surgeon manually positioned the treatment center based on the observed corneal position while maintaining fixation. The corneal vertex coordinates obtained from the Pentacam (OCULUS Optikgeräte GmbH, Wetzlar, Germany) were not entered directly into the surgical system but were documented in the patient chart and used as a reference for centration during surgery.

The VISUMAX 800 procedures were performed during the early implementation phase of the system, before the integration of digital data transfer from IOLMaster 700 (Carl Zeiss Meditec AG, Jena, Germany). Preoperatively, the horizontal and vertical coordinates of the corneal vertex were obtained from Pentacam instead of IOLMaster 700. These coordinates were manually transcribed and entered into the VISUMAX 800 system and used for CentraLign-assisted centration. In addition, reference marks were placed on the cornea with a marking pen before docking using the same criteria as in the VISUMAX 500 group for cyclotorsional adjustment. Thus, the VISUMAX 800 procedures evaluated in this study represent an early, partially manual workflow involving Pentacam-derived corneal vertex coordinates and corneal marking-based cyclotorsional compensation, rather than the currently available digitally integrated workflow.

After femtosecond laser application, the anterior and posterior lenticule planes were dissected using standard surgical instruments, and the lenticule was removed through the small corneal incision. Surgical technique and postoperative management followed the institutional SMILE protocol [7].

### 2.3. AS-OCT Imaging and Measurement of Treatment-Zone Decentration

**Figure 1:**
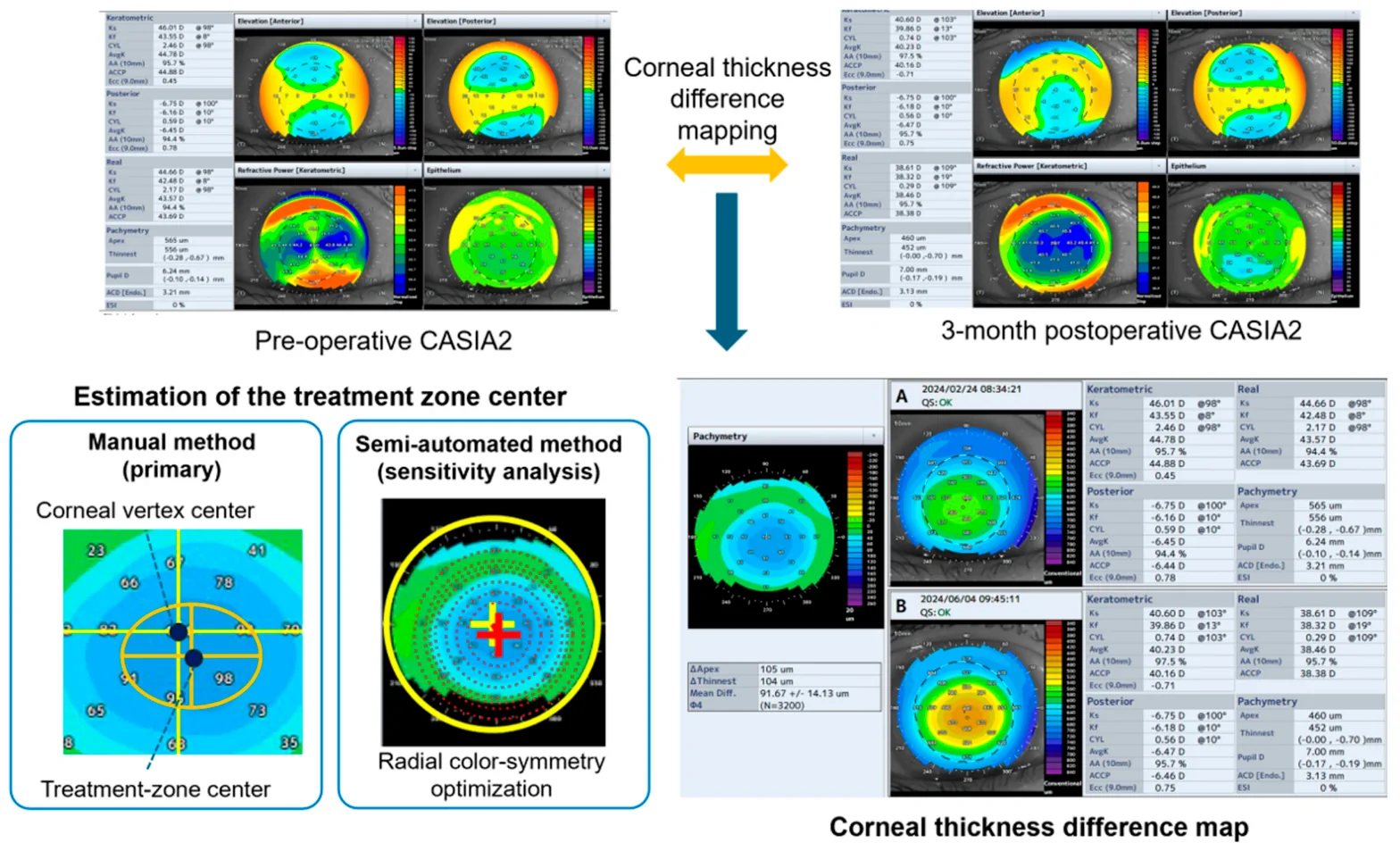
Assessment of treatment-zone decentration using corneal thickness difference maps. Preoperative and 3-month postoperative anterior segment optical coherence tomography images obtained with CASIA2 were used to generate a corneal thickness difference map. The treatment zone was estimated from the spatial distribution of corneal thickness changes. The treatment-zone center was identified, and its displacement relative to the corneal vertex was measured to determine treatment-zone decentration. Manual localization was used for the primary analysis, whereas semi-automated radial color-symmetry localization was used as a sensitivity analysis.

Anterior segment OCT examinations were performed using CASIA2 (Tomey Corporation, Nagoya, Japan) preoperatively and at 3 months after surgery. Preoperative and postoperative corneal thickness maps were used to generate corneal thickness difference maps showing the spatial distribution of postoperative corneal thickness change [8]. The overall measurement workflow is illustrated in Figure 1.

For the primary analysis, the corneal vertex was used as the reference center, and the treatment-zone center was manually identified from the corneal thickness difference map. Horizontal and vertical displacement were recorded relative to the corneal vertex and converted to millimeters using the map scale. Radial decentration was calculated from these coordinates and defined as the primary outcome; absolute horizontal and vertical displacements were secondary outcomes. Manual treatment-zone localization was performed by a single examiner who was masked to the surgical platform. Images were assessed in chronological order rather than randomized order. The treatment-zone center coordinates were recorded at the pixel level and subsequently reviewed twice to verify measurement consistency. Independent measurements by a second examiner were not performed. Instead, a semi-automated localization method was used for additional validation.

As a sensitivity analysis, treatment-zone centers were additionally localized using a semi-automated MATLAB R2026a (MathWorks, Natick, MA, USA) algorithm. The outer boundary of the displayed 10-mm differential pachymetry map was visually confirmed for image-specific calibration [8], after which the treatment-zone center was automatically determined by radial color-symmetry optimization, conceptually based on established radial-symmetry localization methods [9,10]. The a* and b* components of the Commission Internationale de l’Éclairage Lab* (CIELAB) color space were analyzed within a 1.0–4.0 mm annular region, excluding low-saturation and low-intensity pixels and applying robust trimming to reduce the influence of local artifacts. All algorithm parameters were fixed before analysis of the complete cohort. The detailed workflow and complete MATLAB code are provided in Supplementary Figure S1 and Supplementary Code S1.

### 2.4. Statistical Analysis

Continuous variables are presented as mean ± standard deviation and categorical variables as numbers. Because both eyes of the same participant could be included, eye-level comparisons were performed using generalized estimating equations (GEE) with a Gaussian distribution, identity link, exchangeable working correlation structure, and robust standard errors [11]. The primary analysis compared radial decentration between VISUMAX 500 and VISUMAX 800, expressed as the mean difference (VISUMAX 800 minus VISUMAX 500) with a 95% confidence interval (CI). An additional model adjusted for the absolute magnitude of preoperative cylinder, and absolute horizontal and vertical displacements were analyzed as secondary outcomes. Age was compared using Welch’s *t*-test and sex using Fisher’s exact test.

Because the number of independent participants was limited, a participant-level sensitivity analysis was performed by averaging both eyes within each participant and comparing groups using an exact two-sided permutation test, and 95% CIs were calculated using Welch’s t-method. The between-platform analysis was also repeated using semi-automated measurements. Agreement between manual and semi-automated measurements was assessed using two-way absolute-agreement, single-measure intraclass correlation coefficients and Bland–Altman analysis, with bias defined as semi-automated minus manual measurements and 95% limits of agreement calculated as the mean bias ± 1.96 standard deviations of the differences. To account for fellow-eye correlation, 95% CIs for agreement estimates were obtained using 10,000 participant-level cluster bootstrap resamples. No a priori sample-size calculation was performed because the sample was determined by the availability of eligible retrospective cases. All tests were two-sided, with *p* < 0.05 considered statistically significant. Analyses were performed using R software version 4.6.0 (R Foundation for Statistical Computing, Vienna, Austria).

## 3. Results

A total of 44 eyes from 22 participants were included, with 22 eyes from 11 participants in each group. All procedures were completed without intraoperative complications, and no clinically significant postoperative adverse events were documented during the 3-month follow-up period.

Baseline demographic and ocular characteristics are summarized in Table 1. There were no significant differences between the VISUMAX 500 and VISUMAX 800 groups in age, sex, preoperative sphere, cylinder, spherical equivalent, or central corneal thickness (all *p* > 0.05). Preoperative cylinder tended to be greater in the VISUMAX 800 group (−1.73 ± 1.04 D vs. −1.20 ± 0.60 D), although the difference was not statistically significant (*p* = 0.091).

Treatment-zone decentration outcomes are summarized in Table 2. Mean radial decentration was 0.219 ± 0.134 mm in the VISUMAX 500 group and 0.225 ± 0.171 mm in the VISUMAX 800 group. In the GEE analysis accounting for inter-eye correlation, the mean between-group difference was 0.006 mm (95% confidence interval [CI], −0.074 to 0.085; *p* = 0.890). After adjustment for the magnitude of preoperative cylinder, the difference remained nonsignificant (adjusted mean difference, 0.006 mm; 95% CI, −0.082 to 0.094; *p* = 0.888).

**Figure 2:**
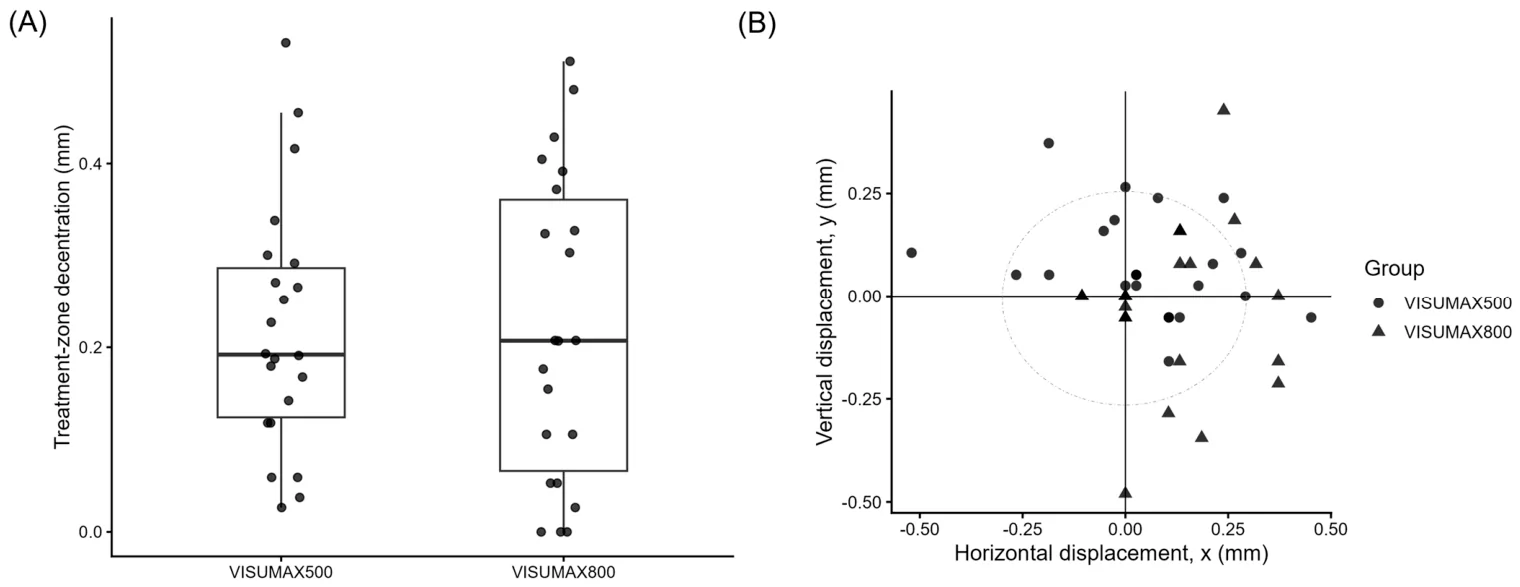
Comparison of treatment-zone decentration between VISUMAX 500 and VISUMAX 800. (**A**) Distribution of radial treatment-zone decentration relative to the corneal vertex. Boxes indicate the interquartile range with the median, and individual points represent eyes. (**B**) Two-dimensional distribution of treatment-zone centers relative to the corneal vertex, defined as the origin (0, 0). Circles and triangles represent eyes treated with VISUMAX 500 and VISUMAX 800, respectively. The dotted circle indicates a radial distance of 0.25 mm from the corneal vertex.

The distribution and spatial pattern of treatment-zone decentration are illustrated in Figure 2. Radial decentration showed substantial overlap between the VISUMAX 500 and VISUMAX 800 groups (Figure 2A). Two-dimensional plotting of horizontal and vertical displacement likewise demonstrated broadly overlapping spatial distributions between the two platforms, without an apparent systematic directional shift (Figure 2B).

Sensitivity analyses of radial decentration are summarized in Table 3. When both eyes were averaged within each participant, mean radial decentration was 0.219 ± 0.090 mm in the VISUMAX 500 group and 0.225 ± 0.108 mm in the VISUMAX 800 group. The participant-level mean difference was 0.0056 mm (95% CI, −0.083 to 0.094), and the exact two-sided permutation test showed no evidence of a between-group difference (*p* = 0.907). Thus, the participant-level analysis was consistent with the primary eye-level GEE analysis.

The analysis was additionally repeated using semi-automated treatment-zone localization. Mean radial decentration was 0.238 ± 0.109 mm for VISUMAX 500 and 0.249 ± 0.167 mm for VISUMAX 800 in the eye-level analysis, with a mean difference of 0.0114 mm (95% CI, −0.063 to 0.086; *p* = 0.763). The corresponding participant-level analysis yielded the same mean difference of 0.0114 mm (95% CI, −0.073 to 0.096; exact permutation *p* = 0.779). These sensitivity analyses yielded findings consistent with the primary manual analysis.

**Figure 3:**
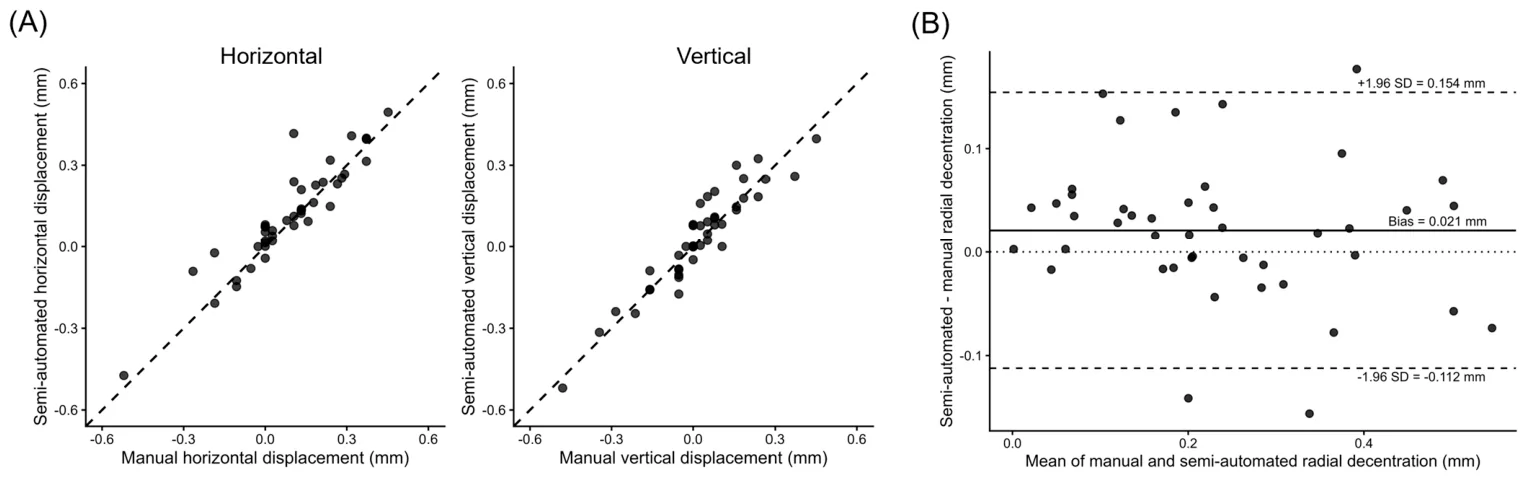
Agreement between manual and semi-automated treatment-zone localization. (**A**) Scatter plots comparing manual and semi-automated measurements of horizontal and vertical treatment-zone displacement. Dashed lines indicate the lines of identity (y = x). (**B**) Bland–Altman plot for radial treatment-zone decentration. Differences were calculated as semi-automated minus manual measurements. The solid horizontal line indicates the mean bias, the dashed lines indicate the 95% limits of agreement, and the dotted line indicates zero difference. Quantitative agreement statistics are provided in Supplementary Table S1.

Agreement between manual and semi-automated treatment-zone localization is shown in Figure 3. Horizontal and vertical displacement measurements were closely distributed around the lines of identity (Figure 3A). Absolute-agreement intraclass correlation coefficients (ICCs) were 0.916 for horizontal displacement, 0.934 for vertical displacement, and 0.885 for radial decentration (Supplementary Table S1). For radial decentration, Bland–Altman analysis showed a small positive bias of 0.021 mm (95% CI, 0.003 to 0.041) for the semi-automated method relative to the manual method, with 95% limits of agreement from −0.112 to +0.154 mm (Figure 3B).

## 4. Discussion

In this study, treatment-zone decentration after SMILE did not differ significantly between VISUMAX 500 and VISUMAX 800 when assessed relative to the corneal vertex using postoperative corneal thickness difference maps. Mean radial decentration was 0.219 mm with VISUMAX 500 and 0.225 mm with VISUMAX 800, and the between-group difference remained minimal after accounting for inter-eye correlation and adjusting for preoperative astigmatism. Horizontal and vertical displacement also showed no significant between-group differences. Participant-level and semi-automated sensitivity analyses yielded consistent findings. These results indicate that, under the surgical conditions evaluated in this study, CentraLign-assisted centration with VISUMAX 800 was not associated with a statistically significant reduction in treatment-zone decentration compared with fixation-based manual centration using VISUMAX 500.

The technical evolution of keratorefractive lenticule extraction has increasingly emphasized accurate treatment centration and control of cyclotorsional or astigmatic-axis misalignment, particularly as the procedure has expanded to eyes with greater astigmatism [12,13]. VISUMAX 800 was developed within this broader progression, incorporating assisted centration and cyclotorsional alignment functions intended to improve treatment placement and reproducibility [5].

The surgical context should be considered when interpreting these findings. VISUMAX 500 had been used routinely for many years, and the surgeon was highly experienced with fixation-based manual centration, whereas VISUMAX 800 was evaluated during its early implementation period. The small magnitude of decentration observed with both platforms suggests that experienced manual centration with VISUMAX 500 may already achieve a high level of treatment-zone alignment, potentially limiting the additional improvement detectable with assisted centration. At the same time, platform-specific workflow familiarity and a learning-curve effect with VISUMAX 800 cannot be excluded. Because the present study was not designed as an equivalence or noninferiority trial, the absence of a statistically significant difference should not be interpreted as evidence of equivalent centration performance [14].

The VISUMAX 800 workflow evaluated in this study also differed from the contemporary fully integrated workflow. Corneal vertex coordinates were obtained from Pentacam and manually transferred to the VISUMAX 800 system for CentraLign-assisted centration, whereas contemporary workflows use digitally transferred coordinates from the IOLMaster 700 [15]. Although Pentacam and IOLMaster 700 provide related measurements of the spatial relationship between the corneal vertex and pupil center, systematic device-specific differences limit their direct interchangeability [16,17]. Accordingly, the present findings should be interpreted as reflecting an early, partially manual CentraLign workflow rather than the current fully integrated VISUMAX 800 workflow. Cyclotorsional adjustment was additionally performed using corneal ink markings rather than digital iris registration. Manual transcription of corneal vertex coordinates may increase workflow complexity and introduce potential transcription errors, limiting its scalability compared with automated digital data transfer.

Our findings differ from those of Qian et al. [6], who reported lower treatment-zone decentration with VISUMAX 800 than with VISUMAX 500, mainly due to reduced vertical displacement. This discrepancy may reflect differences in study design, sample size, platform-specific centration techniques, and measurement methodology, because their prospective matched study used tangential topography difference maps, whereas we used AS-OCT corneal thickness difference maps.

The magnitude of decentration observed in the present study was within the range reported in previous SMILE studies, although direct comparison should be made cautiously because several different methods have been used to define and measure treatment-zone centration. Previous investigators have assessed decentration using tangential curvature difference maps, pachymetric difference maps, postoperative topography, or intraoperative video images, with reference points including the pupil center, coaxially sighted corneal light reflex, and corneal vertex [18,19,20]. For example, a study using tangential curvature difference maps reported a mean corneal vertex-referenced centration offset of approximately 0.20 mm after SMILE, similar to the values observed in our cohort [21]. More recent work also emphasizes that estimates of treatment-zone decentration depend substantially on both the imaging modality and the method used to identify the optical-zone center [22]. In the present study, treatment-zone localization was based on preoperative-to-postoperative CASIA2 corneal thickness difference maps with the corneal vertex as the reference center. However, difference-map-based measurements have inherent limitations because refractive surgery may alter corneal geometry and the reference axis between preoperative and postoperative acquisitions, potentially affecting image registration and the apparent position of the treatment zone [23]. Therefore, the measured decentration may reflect both true treatment-zone displacement and measurement variability related to postoperative geometric and registration changes.

To address uncertainty associated with manual localization, we additionally applied a semi-automated radial color-symmetry method. Absolute-agreement ICCs ranged from 0.885 to 0.934, and the semi-automated method showed only a small positive bias in radial decentration (+0.021 mm). Nevertheless, the limits of agreement (−0.112 to +0.154 mm) indicate that individual-eye localization remains subject to measurement variability. Importantly, repeating the between-platform analysis using semi-automated measurements did not materially alter the study findings. Further reduction in observer dependence may be achievable through fully automated image-analysis approaches. Deep learning and transfer learning have enabled automated feature localization and classification across diverse medical imaging tasks, including dermoscopic imaging and musculoskeletal MRI [24,25]. Similar approaches could be explored for automated treatment-zone localization in anterior segment OCT when larger annotated datasets become available.

Preoperative astigmatism also warrants consideration. The VISUMAX 800 group tended to have greater preoperative cylinder than the VISUMAX 500 group, although this difference was not statistically significant after accounting for inter-eye correlation. A previous study reported a significant association between anterior keratometric astigmatism and treatment-zone decentration after SMILE [2]. In the present analysis, however, adjustment for the magnitude of preoperative cylinder resulted in essentially no change in the estimated difference between platforms, suggesting that the observed similarity in centration was not explained by this baseline imbalance.

The small number of independent participants limited the ability to detect modest between-platform differences. Furthermore, robust sandwich variance estimates from GEE may be unstable with a limited number of independent clusters. We therefore performed a participant-level exact permutation analysis, which yielded results consistent with the primary GEE analysis. An approximate sensitivity calculation based on the observed participant-level standard deviations and 11 participants per group indicated that a conventional two-sample comparison would have 80% power to detect a mean difference of approximately 0.125 mm at a two-sided significance level of 0.05. This estimate should be interpreted as illustrative rather than as the statistical power of the primary GEE analysis. The 95% CI for the primary estimate ranged from −0.074 to +0.085 mm, indicating that between-platform differences of approximately 0.08 mm in either direction remained compatible with the observed data. Because no established threshold defines a clinically meaningful between-platform difference in mean radial decentration, an equivalence margin was not prespecified. Therefore, the absence of a statistically significant difference should not be interpreted as evidence of equivalence between the two platforms [14].

VISUMAX 800 cases were obtained during the early implementation period, whereas the surgeon had extensive prior experience with VISUMAX 500. Therefore, platform-specific workflow familiarity and a potential learning-curve effect cannot be excluded. However, the two platforms were used during substantially overlapping calendar periods rather than as strictly sequential cohorts. A formal learning-curve analysis was not considered reliable because the study cohort was selected according to the availability of paired AS-OCT examinations, with intervening surgical cases excluded when eligible follow-up imaging was unavailable. In addition, the available imaging dates did not reliably reflect the chronological order of surgery. Consequently, cumulative procedure order could not be reconstructed with sufficient accuracy for a meaningful learning-curve analysis.

Several additional limitations should be acknowledged. First, the retrospective, single-center design may limit the generalizability of the findings. Because platform selection was based on patient preference under different pricing policies rather than random assignment, selection bias and unmeasured confounding related to socioeconomic factors or patient characteristics cannot be excluded. Second, although manual localization was performed by a platform-masked examiner and pixel-level measurements were reviewed twice, formal intra-observer repeatability and independent interobserver reproducibility were not assessed. To evaluate potential measurement uncertainty, we performed an additional semi-automated localization analysis. Agreement between the manual and semi-automated methods was high, and the between-platform analysis using semi-automated measurements yielded findings consistent with the primary analysis. Nevertheless, the Bland–Altman limits of agreement indicate that some localization uncertainty remains at the individual-eye level. Third, the image-derived coordinates required uniform spatial calibration before conversion to millimeters. Although the same procedure was applied to both groups and therefore should not preferentially bias either platform, measurement error remains possible. Fourth, both eyes were included for many participants; however, within-subject correlation was accounted for using GEE, and the findings were consistent in the participant-level sensitivity analysis. Fifth, postoperative visual, refractive, and higher-order aberration data were not uniformly available for the complete cohort. We therefore did not perform an available-case comparison, which could have introduced selection bias. Accordingly, the present findings are limited to anatomical treatment-zone centration and should not be interpreted as demonstrating equivalent functional or visual outcomes between the two platforms. In addition, treatment-zone decentration was assessed only at 3 months postoperatively, and its longer-term stability could not be evaluated. Finally, all procedures were performed by a single highly experienced surgeon. This reduced surgeon-related variability and strengthened the internal comparison between platforms but may limit generalizability, particularly to surgeons with less experience in fixation-based manual centration.

## 5. Conclusions

In conclusion, treatment-zone decentration relative to the corneal vertex did not differ significantly between VISUMAX 500 and VISUMAX 800 in this small single-surgeon cohort. Participant-level and semi-automated sensitivity analyses yielded consistent findings. These results apply to the early, partially manual VISUMAX 800 workflow evaluated in this study and should not be interpreted as demonstrating equivalence between platforms or as directly representing contemporary fully integrated VISUMAX 800 workflows. Larger prospective studies incorporating functional visual outcomes are warranted.

## Figures and Tables

**Table 1:** Baseline demographic and ocular characteristics.

Characteristic	VISUMAX 500	VISUMAX 800	*p* Value
Participants, n	11	11	
Eyes, n	22	22	
Age, years	23.09 ± 3.30	25.55 ± 4.72	0.175
Sex, M/F	5/6	6/5	1.000
Sphere, D	−3.69 ± 1.01	−3.66 ± 0.62	0.917
Cylinder, D	−1.20 ± 0.60	−1.73 ± 1.04	0.091
Spherical equivalent, D	−4.30 ± 1.13	−4.52 ± 0.83	0.556
Central corneal thickness, μm	543.09 ± 14.87	549.41 ± 26.83	0.479

Footnotes: Values are presented as mean ± standard deviation unless otherwise indicated. Age and sex were compared at the participant level. Eye-level ocular variables were compared using generalized estimating equations to account for correlation between fellow eyes. Sex was compared using Fisher’s exact test. Abbreviations: D, diopters; M, male; F, female.

**Table 2:** Treatment-zone decentration outcomes according to VISUMAX platform.

Measurement	VISUMAX 500, mm	VISUMAX 800, mm	Mean Difference, mm	95% CI, mm	*p* Value
Absolute horizontal displacement *	0.159 ± 0.141	0.144 ± 0.129	−0.0151	−0.077 to +0.047	0.634
Absolute vertical displacement *	0.110 ± 0.097	0.138 ± 0.154	0.0288	−0.050 to +0.108	0.475
Radial decentration *	0.219 ± 0.134	0.225 ± 0.171	0.0056	−0.074 to +0.085	0.890
Radial decentration, adjusted for preoperative cylinder ^†^	0.219 ± 0.134	0.225 ± 0.171	0.0063	−0.082 to +0.094	0.888

Footnotes: Values are presented as mean ± standard deviation. * Mean differences represent VISUMAX 800 minus VISUMAX 500. *p* values and 95% confidence intervals were estimated using generalized estimating equations accounting for correlation between fellow eyes. ^†^ Adjusted for the magnitude of preoperative cylinder. Abbreviations: CI, confidence interval.

**Table 3:** Sensitivity analyses of radial treatment-zone decentration.

Analysis	VISUMAX 500, mm	VISUMAX 800, mm	Mean Difference, mm	95% CI, mm	*p* Value
Manual measurement: eye-level GEE	0.219 ± 0.134	0.225 ± 0.171	0.0056	−0.074 to +0.085	0.890
Manual measurement: participant-level bilateral mean	0.219 ± 0.090	0.225 ± 0.108	0.0056	−0.083 to +0.094	0.907
Semi-automated measurement: eye-level GEE	0.238 ± 0.109	0.249 ± 0.167	0.0114	−0.063 to +0.086	0.763
Semi-automated measurement: participant-level bilateral mean	0.238 ± 0.060	0.249 ± 0.117	0.0114	−0.073 to +0.096	0.779

Footnote: Values are presented as mean ± standard deviation. Mean differences were calculated as VISUMAX 800 minus VISUMAX 500. Eye-level analyses were performed using generalized estimating equations with a Gaussian distribution, identity link, exchangeable working correlation structure, and robust standard errors to account for correlation between fellow eyes. For participant-level sensitivity analyses, measurements from both eyes were averaged within each participant, and between-group differences were assessed using an exact two-sided permutation test based on all 705,432 possible allocations of 22 participants into two groups of 11. Participant-level confidence intervals were calculated using Welch’s t-method. CI, confidence interval; GEE, generalized estimating equation.

## Data Availability

The complete clinical dataset is not publicly available because of patient privacy and institutional ethical restrictions. The MATLAB code for semi-automated treatment-zone localization and de-identified representative CASIA2 differential pachymetry maps for reproducibility testing are publicly available at https://github.com/TaeKeunToo/Detect_treatment_center (accessed on 6 October 2026). Additional study data may be available from the corresponding author upon reasonable request and subject to applicable ethical and institutional restrictions.

## Supplementary Materials

The following supporting information can be downloaded at: https://www.mdpi.com/article/10.3390/jcm15207851/s1, Supplementary Table S1. Agreement between manual and semi-automated measurements. Supplementary Figure S1. Semi-automated radial color-symmetry method for treatment-zone center localization. (A) Differential pachymetry map used as the input image. (B) The outer boundary of the 10-mm differential pachymetry map was initialized using standardized relative image coordinates and visually confirmed by the operator, with adjustment when necessary. The confirmed radius was assigned a value of 5 mm for image-specific spatial calibration. (C) Candidate treatment-zone centers were evaluated within an annular region from 1.0 to 4.0 mm using concentric radial bins and CIELAB a* and b* color information. The treatment-zone center was defined as the location minimizing robust within-ring color variability after exclusion of low-saturation and low-intensity pixels and trimming of local outliers. (D) Horizontal and vertical displacement were calculated relative to the reference center. Supplementary Code S1. MATLAB code for semi-automated treatment-zone center localization from CASIA2 differential pachymetry maps.

## Abbreviations

The following abbreviations are used in this manuscript:

AS-OCT	Anterior segment optical coherence tomography
CI	Confidence interval
D	Diopter
GEE	Generalized estimating equation
ICC	Intraclass correlation coefficient
SMILE	Small-incision lenticule extraction
