# Supplementary material for: Comparison of Treatment Zone Decentration Between VISUMAX 500 and VISUMAX 800 Keratorefractive Lenticule Extraction Using Anterior Segment Optical Coherence Tomography
Source: J Clin Med. 2026 Oct 12;15(20):7851. doi: 10.3390/jcm15207851 (PMC13705058; doi:10.3390/jcm15207851)
Supplement: Supplementary file 1 [file jcm-15-07851-s001.zip › jcm-4590030-supplementary.pdf]

Supplementary Materials

Supplementary Table S1. Agreement between manual and semi-automated measurements

Measurement	No. of eyes	ICC (95% CI)	Mean bias, mm (95% CI)	95% limits of agreement, mm
Horizontal displacement	44	0.916 (0.811 to 0.968)	+0.027 (+0.006 to +0.050)	−0.113 to +0.167
Vertical displacement	44	0.934 (0.856 to 0.964)	+0.005 (−0.012 to +0.023)	−0.123 to +0.134
Radial decentration	44	0.885 (0.787 to 0.943)	+0.021 (+0.003 to +0.041)	−0.112 to +0.154

Footnote: Bias was defined as the semi-automated measurement minus the manual measurement. ICCs represent two-way absolute-agreement, single-measure estimates. Because both eyes of each participant were included, 95% confidence intervals were obtained using 10,000 participant-level cluster bootstrap resamples, with both eyes of each participant resampled together. Limits of agreement were calculated as the mean bias ±1.96 standard deviations of the between-method differences.

Abbreviations: ICC, intraclass correlation coefficient; CI, confidence interval.

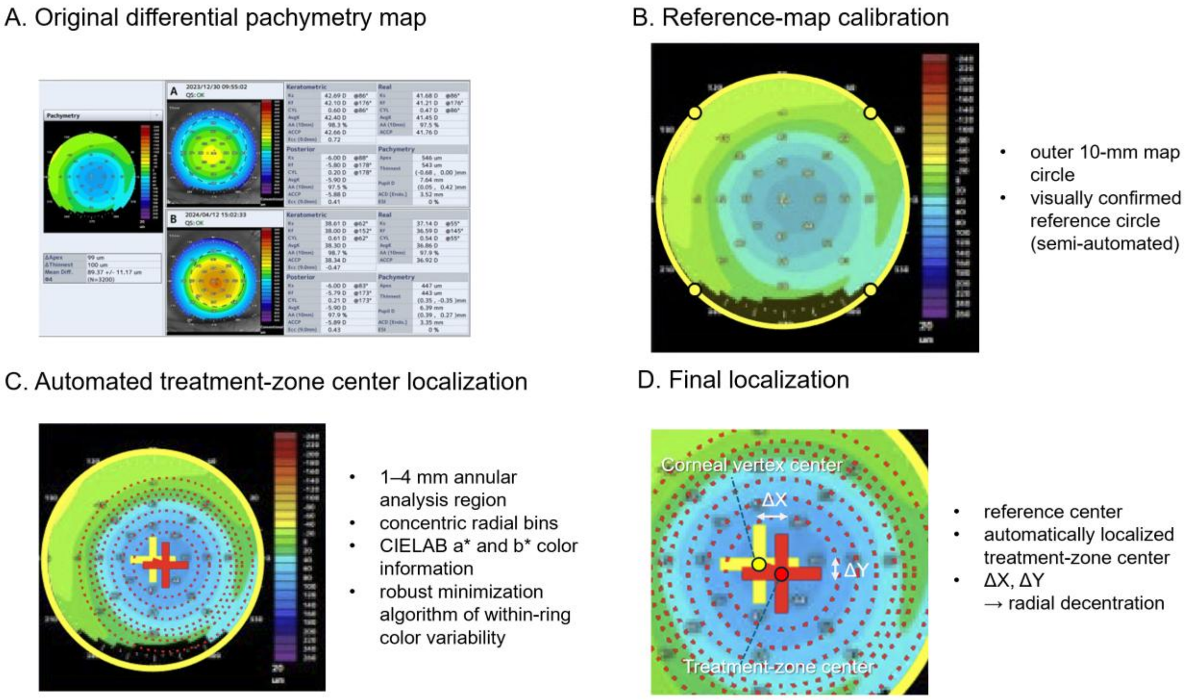

Supplementary Figure S1. Semi-automated radial color-symmetry method for treatment-zone center localization. (A) Differential pachymetry map used as the input image. (B) The outer boundary of the 10-mm differential pachymetry map was initialized using standardized relative image coordinates and visually confirmed by the operator, with adjustment when necessary. The confirmed radius was assigned a value of 5 mm for image-specific spatial calibration. (C) Candidate treatment-zone centers were evaluated within an annular region from 1.0 to 4.0 mm using concentric radial bins and CIELAB a* and b* color information. The treatment-zone center was defined as the location minimizing robust within-ring color variability after exclusion of low-saturation and low-intensity pixels and trimming of local outliers. (D) Horizontal and vertical displacement were calculated relative to the reference center.

Supplementary Code S1. MATLAB code for semi-automated treatment-zone center localization from CASIA2 differential pachymetry maps.

The code performs image-specific spatial calibration using the outer boundary of the displayed 10-mm differential pachymetry map, which is initialized at standardized relative image coordinates and visually confirmed or manually adjusted by the operator when necessary. After calibration, treatment-zone center localization is performed automatically using a radial color-symmetry optimization algorithm. CIELAB a* and b* color information is analyzed within concentric radial bins spanning 1.0–4.0 mm from the reference center, with exclusion of low-saturation and low-intensity pixels and robust trimming of local outliers. The candidate location minimizing within-ring color variability is selected as the treatment-zone center. The code outputs horizontal displacement, vertical displacement, radial decentration, image-specific calibration parameters, and quality-control images. All algorithm parameters were fixed before analysis of the complete study cohort. For transparency and reproducibility, the complete MATLAB implementation together with de-identified representative CASIA2 differential pachymetry maps is publicly available at https://github.com/TaeKeunToo/Detect_treatment_center.

```
%% ========================================================================
% CASIA2 BATCH SEMI-AUTOMATED TREATMENT-ZONE CENTER ANALYSIS
%
% Reference center:
%     User visually confirms/adjusts the OUTER 10-mm pachymetry circle.
%
% Treatment center:
%     Automatically determined by maximizing radial color symmetry
%     of the differential pachymetry map.
%
% Workflow:
%     1. Select folder
%     2. Each image opens sequentially
%     3. Yellow reference circle appears automatically
%     4. If correct: double-click the circle without moving it
%        If incorrect: move/resize, then double-click
%     5. Treatment-zone center is calculated automatically
%     6. QC image is saved
%     7. Next image automatically opens
%     8. Final CSV/XLSX summary is saved
%
% Assumption:
%     Outer pachymetry map diameter = 10.0 mm
%
% Coordinate convention:
%     X > 0 : right
%     X < 0 : left
%     Y > 0 : superior
%     Y < 0 : inferior
%
% Requires:
%     Image Processing Toolbox
%
% ========================================================================

clear;
close all;
clc;


%% ========================================================================
% SETTINGS
% ========================================================================

OUTER_DIAMETER_MM = 10.0;
OUTER_RADIUS_MM     = OUTER_DIAMETER_MM / 2;

% Radial region used for automated center estimation
ANALYSIS_R_MIN_MM = 1.0;
ANALYSIS_R_MAX_MM = 4.0;

% Maximum allowed center displacement
MAX_OFFSET_MM = 1.20;

% Radial bin width
RADIAL_BIN_MM = 0.12;

% Exclude low-saturation pixels such as white/gray labels
MIN_SATURATION = 0.25;

% Exclude very dark pixels
MIN_VALUE = 0.12;
```

```
% Robust trimming inside each radial ring
TRIM_PERCENT = 85;

% Optimization tolerance
OPT_TOL_MM = 0.002;

% Coarse search step
COARSE_STEP_MM = 0.10;

% QC display radii
QC_RADII_MM = [1.5 2.0 2.5 3.0 3.25 3.5 4.0];


%% ========================================================================
% SELECT FOLDER
% ========================================================================

folder = uigetdir(pwd, ...
    'Select folder containing CASIA2 images');

if isequal(folder,0)
    error('No folder selected.');
end


%% ========================================================================
% CREATE OUTPUT FOLDER
% ========================================================================

qcFolder = fullfile(folder,'CASIA2_QC');

if ~exist(qcFolder,'dir')
    mkdir(qcFolder);
end


%% ========================================================================
% FIND IMAGE FILES
% ========================================================================

files = [
    dir(fullfile(folder,'*.jpg'))
    dir(fullfile(folder,'*.JPG'))
    dir(fullfile(folder,'*.jpeg'))
    dir(fullfile(folder,'*.JPEG'))
    dir(fullfile(folder,'*.png'))
    dir(fullfile(folder,'*.PNG'))
    ];


% Exclude previously generated QC images
keep = true(numel(files),1);

for i = 1:numel(files)

    nm = lower(files(i).name);

    if contains(nm,'_qc') || ...
        contains(nm,'semi_auto') || ...
        contains(nm,'semiauto')

          keep(i) = false;
    end

end

files = files(keep);


if isempty(files)
    error('No JPG/PNG images found.');
end


% Alphabetical sorting
[~,idx] = sort(lower({files.name}));
files = files(idx);

nFiles = numel(files);


fprintf('\n');
fprintf('==============================================================\n');
```

```
fprintf('CASIA2 BATCH SEMI-AUTOMATED ANALYSIS\n');
fprintf('Number of images: %d\n',nFiles);
fprintf('==============================================================\n\n');


%% ============================================================================
% RESULT STORAGE
% ============================================================================

results = cell(nFiles,20);


%% ============================================================================
% MAIN LOOP
% ============================================================================

for i = 1:nFiles

    filename = files(i).name;
    filepath = fullfile(folder,filename);

    fprintf('\n--------------------------------------------------------------\n');
    fprintf('[%d/%d] %s\n',i,nFiles,filename);
    fprintf('--------------------------------------------------------------\n');


    try

        %% -----------------------------------------------------------------
        % READ IMAGE
        % -----------------------------------------------------------------

        I = imread(filepath);

        if size(I,3) == 1
            I = repmat(I,[1 1 3]);
        end

        [H,W,~] = size(I);


        %% ==================================================================
        % STEP 1
        % USER-CONFIRMED 10-mm REFERENCE CIRCLE
        %
        % IMPORTANT:
        % No automatic color segmentation is used here.
        %
        % Initial circle is based only on standardized relative position.
        % ==================================================================

        initialCenter = [ ...
            0.16 * W, ...
            0.46 * H];

        initialRadius = ...
            0.13 * H;


        fRef = figure( ...
            'Color','w', ...
            'Name',sprintf('Reference circle %d/%d',i,nFiles), ...
            'NumberTitle','off');

        try
            fRef.WindowState = 'maximized';
        catch
        end


        imshow(I);
        hold on;


        hCircle = drawcircle( ...
            'Center',initialCenter, ...
            'Radius',initialRadius, ...
            'Color','yellow', ...
            'LineWidth',2.5);


        title({ ...
            sprintf('[%d/%d] %s',i,nFiles), ...
            'Adjust yellow circle to the OUTER 10-mm pachymetry map', ...
```

```matlab
        'If already correct, do not move it', ...
        'DOUBLE-CLICK the circle when finished'}, ...
        'Interpreter','none');


% Wait until user double-clicks ROI
wait(hCircle);


% Read final selected circle
refCenter = hCircle.Center;

cx_ref = refCenter(1);
cy_ref = refCenter(2);

R_ref_px = hCircle.Radius;


%% ---------------------------------------------------------------
% RECORD WHETHER USER ADJUSTED REFERENCE CIRCLE
% ---------------------------------------------------------------

centerAdjustment_px = hypot( ...
        cx_ref-initialCenter(1), ...
        cy_ref-initialCenter(2));

radiusAdjustment_px = ...
        abs(R_ref_px-initialRadius);


% Consider >1 pixel change as adjusted
wasAdjusted = ...
        centerAdjustment_px > 1 || ...
        radiusAdjustment_px > 1;


close(fRef);


%% ---------------------------------------------------------------
% IMAGE-SPECIFIC SCALE
% ---------------------------------------------------------------

mm_per_px = ...
        OUTER_RADIUS_MM / R_ref_px;

px_per_mm = ...
        1 / mm_per_px;


fprintf('Reference center : %.2f, %.2f px\n', ...
        cx_ref,cy_ref);

fprintf('Reference radius : %.2f px\n', ...
        R_ref_px);

fprintf('Scale            : %.5f mm/px\n', ...
        mm_per_px);

fprintf('Reference adjusted: %d\n', ...
        wasAdjusted);


%% ===============================================================
% STEP 2
% PREPARE COLOR DATA
% ===============================================================

Lab = rgb2lab(I);

A = double(Lab(:,:,2));
B = double(Lab(:,:,3));

% Mild smoothing to reduce JPEG noise
A = imgaussfilt(A,1.0);
B = imgaussfilt(B,1.0);


HSV = rgb2hsv(I);

S = double(HSV(:,:,2));
V = double(HSV(:,:,3));
```

```
[Xpix,Ypix] = meshgrid(1:W,1:H);


%% ================================================================
% STEP 3
% DEFINE ANALYSIS MASK
%
% Only pixels within 1-4 mm from reference center are used.
% Color bar cannot enter because it lies outside the reference map.
% ================================================================

Rmm_ref = ...
    hypot( ...
    Xpix-cx_ref, ...
    Ypix-cy_ref) ...
    * mm_per_px;


analysisMask = ...
    Rmm_ref >= ANALYSIS_R_MIN_MM & ...
    Rmm_ref <= ANALYSIS_R_MAX_MM & ...
    S >= MIN_SATURATION & ...
    V >= MIN_VALUE;


xData = double(Xpix(analysisMask));
yData = double(Ypix(analysisMask));

aData = A(analysisMask);
bData = B(analysisMask);


numberPixels = numel(xData);


if numberPixels < 500
    error('Too few usable pixels for treatment-center analysis.');
end


%% ================================================================
% STEP 4
% DEFINE RADIAL-SYMMETRY OBJECTIVE
% ================================================================

objective = @(offset) radialSymmetryCost( ...
    offset, ...
    cx_ref, ...
    cy_ref, ...
    xData, ...
    yData, ...
    aData, ...
    bData, ...
    mm_per_px, ...
    ANALYSIS_R_MIN_MM, ...
    ANALYSIS_R_MAX_MM, ...
    RADIAL_BIN_MM, ...
    TRIM_PERCENT, ...
    MAX_OFFSET_MM);


%% ================================================================
% STEP 5
% COARSE GRID SEARCH
% ================================================================

offsetValues = ...
    -MAX_OFFSET_MM:COARSE_STEP_MM:MAX_OFFSET_MM;


bestCost = Inf;
bestOffset = [0 0];


for dx = offsetValues

    for dy = offsetValues

        c = objective([dx dy]);

        if c < bestCost

            bestCost = c;
            bestOffset = [dx dy];
```

```
            end

        end

    end


    fprintf('Coarse center         : X=%+.3f, Y=%+.3f mm\n', ...
        bestOffset(1), ...
        bestOffset(2));


    %% ================================================================
    % STEP 6
    % CONTINUOUS FINE OPTIMIZATION
    % ================================================================

    options = optimset( ...
        'Display','off', ...
        'TolX',OPT_TOL_MM, ...
        'TolFun',1e-6, ...
        'MaxIter',500);


    [bestOffset,bestCost] = ...
        fminsearch( ...
        objective, ...
        bestOffset, ...
        options);


    %% ================================================================
    % STEP 7
    % FINAL TREATMENT CENTER
    % ================================================================

    dx_mm = bestOffset(1);
    dy_mm = bestOffset(2);


    radial_mm = ...
        hypot(dx_mm,dy_mm);


    cx_treat = ...
        cx_ref + ...
        dx_mm/mm_per_px;


    % Image Y increases downward.
    % Physical positive Y = superior.
    cy_treat = ...
        cy_ref - ...
        dy_mm/mm_per_px;


    %% ================================================================
    % STEP 8
    % SYMMETRY IMPROVEMENT
    % ================================================================

    referenceCost = ...
        objective([0 0]);


    symmetryImprovement = ...
        100 * ...
        (referenceCost-bestCost) ...
        / referenceCost;


    %% ================================================================
    % STEP 9
    % BASIC QC FLAG
    % ================================================================

    QC = "PASS";
    QCReason = "none";


    if radial_mm > 0.95*MAX_OFFSET_MM

        QC = "CHECK";
```

```
        QCReason = "center near search limit";

    end


    if symmetryImprovement < 0.5

        QC = "CHECK";

        if QCReason == "none"
            QCReason = "low symmetry improvement";
        else
            QCReason = ...
                QCReason + "; low symmetry improvement";
        end

    end


    %% ==============================================================
    % STEP 10
    % QC FIGURE
    % ==============================================================

    fQC = figure( ...
        'Color','w', ...
        'Visible','off');


    imshow(I);
    hold on;


    theta = linspace(0,2*pi,500);


    % -------------------------------------------------------
    % Yellow outer reference circle
    % -------------------------------------------------------

    plot( ...
        cx_ref + R_ref_px*cos(theta), ...
        cy_ref + R_ref_px*sin(theta), ...
        'y-', ...
        'LineWidth',2.5);


    % Reference center
    plot( ...
        cx_ref, ...
        cy_ref, ...
        'y+', ...
        'MarkerSize',18, ...
        'LineWidth',3);


    % -------------------------------------------------------
    % Red automated treatment center
    % -------------------------------------------------------

    plot( ...
        cx_treat, ...
        cy_treat, ...
        'r+', ...
        'MarkerSize',18, ...
        'LineWidth',3);


    % Decentration vector
    plot( ...
        [cx_ref cx_treat], ...
        [cy_ref cy_treat], ...
        'r-', ...
        'LineWidth',2);


    % -------------------------------------------------------
    % QC concentric circles centered on automated center
    %
    % These circles are VISUAL REFERENCES ONLY.
    % They do not determine the automated center.
    % -------------------------------------------------------

    for rmm = QC_RADII_MM
```

```
        rp = rmm / mm_per_px;

        plot( ...
            cx_treat + rp*cos(theta), ...
            cy_treat + rp*sin(theta), ...
            'r:', ...
            'LineWidth',0.8);

    end


    title(sprintf( ...
        '%s | X=%+.3f mm | Y=%+.3f mm | Radial=%.3f mm | %s', ...
        filename, ...
        dx_mm, ...
        dy_mm, ...
        radial_mm, ...
        QC), ...
        'Interpreter','none');


    hold off;


    %% ----------------------------------------------------------------
    % SAVE QC IMAGE
    % ----------------------------------------------------------------

    [~,baseName,~] = ...
        fileparts(filename);


    qcFilename = ...
        fullfile( ...
        qcFolder, ...
        [baseName '_QC.png']);


    exportgraphics( ...
        fQC, ...
        qcFilename, ...
        'Resolution',200);


    close(fQC);


    %% ================================================================
    % STEP 11
    % STORE RESULTS
    % ================================================================

    results{i,1} = filename;

    results{i,2} = W;
    results{i,3} = H;

    results{i,4} = initialCenter(1);
    results{i,5} = initialCenter(2);
    results{i,6} = initialRadius;

    results{i,7} = cx_ref;
    results{i,8} = cy_ref;
    results{i,9} = R_ref_px;

    results{i,10} = wasAdjusted;

    results{i,11} = mm_per_px;

    results{i,12} = cx_treat;
    results{i,13} = cy_treat;

    results{i,14} = dx_mm;
    results{i,15} = dy_mm;
    results{i,16} = radial_mm;

    results{i,17} = symmetryImprovement;

    results{i,18} = numberPixels;

    results{i,19} = char(QC);
    results{i,20} = char(QCReason);
```

```
            fprintf('\n');
            fprintf('FINAL RESULT\n');
            fprintf('X          = %+0.3f mm\n',dx_mm);
            fprintf('Y          = %+0.3f mm\n',dy_mm);
            fprintf('Radial = %0.3f mm\n',radial_mm);
            fprintf('Symmetry improvement = %.2f %%\n', ...
                symmetryImprovement);
            fprintf('QC = %s\n',QC);


        catch ME

            fprintf('\n*** FAILED: %s\n',ME.message);


            results{i,1} = filename;

            for j = 2:18
                results{i,j} = NaN;
            end

            results{i,19} = 'FAILED';
            results{i,20} = ME.message;


            % Close accidental remaining figures
            close all;

        end

end


%% ======================================================================
% CREATE FULL RESULT TABLE
% ======================================================================

variableNames = { ...
    'Filename', ...
    'ImageWidth_px', ...
    'ImageHeight_px', ...
    'InitialReferenceCenterX_px', ...
    'InitialReferenceCenterY_px', ...
    'InitialReferenceRadius_px', ...
    'FinalReferenceCenterX_px', ...
    'FinalReferenceCenterY_px', ...
    'FinalReferenceRadius_px', ...
    'ReferenceCircleAdjusted', ...
    'Scale_mm_per_px', ...
    'TreatmentCenterX_px', ...
    'TreatmentCenterY_px', ...
    'HorizontalDecentration_mm', ...
    'VerticalDecentration_mm', ...
    'RadialDecentration_mm', ...
    'SymmetryImprovement_percent', ...
    'PixelsAnalyzed', ...
    'QC', ...
    'QCReason'};


T = cell2table( ...
    results, ...
    'VariableNames',variableNames);


%% ======================================================================
% SAVE FULL RESULTS
% ======================================================================

csvFile = ...
    fullfile( ...
    folder, ...
    'CASIA2_batch_results.csv');


xlsxFile = ...
    fullfile( ...
    folder, ...
    'CASIA2_batch_results.xlsx');


writetable(T,csvFile);
writetable(T,xlsxFile);
```

```
%% =====================================================================
% CREATE SIMPLE ANALYSIS TABLE
% =====================================================================

T_simple = T(:,{ ...
    'Filename', ...
    'HorizontalDecentration_mm', ...
    'VerticalDecentration_mm', ...
    'RadialDecentration_mm', ...
    'ReferenceCircleAdjusted', ...
    'QC', ...
    'QCReason'});


simpleXlsx = ...
    fullfile( ...
    folder, ...
    'CASIA2_decentration_summary.xlsx');


simpleCsv = ...
    fullfile( ...
    folder, ...
    'CASIA2_decentration_summary.csv');


writetable(T_simple,simpleXlsx);
writetable(T_simple,simpleCsv);


%% =====================================================================
% FINAL SUMMARY
% =====================================================================

fprintf('\n');
fprintf('============================================================\n');
fprintf('BATCH ANALYSIS COMPLETE\n');
fprintf('============================================================\n');

fprintf('Total images: %d\n',height(T));

fprintf('PASS     : %d\n', ...
    sum(strcmp(T.QC,'PASS')));

fprintf('CHECK   : %d\n', ...
    sum(strcmp(T.QC,'CHECK')));

fprintf('FAILED : %d\n', ...
    sum(strcmp(T.QC,'FAILED')));


validAdjusted = false(height(T),1);

for i = 1:height(T)

    try
        validAdjusted(i) = ...
            logical(T.ReferenceCircleAdjusted{i});
    catch
        try
            validAdjusted(i) = ...
                logical(T.ReferenceCircleAdjusted(i));
        catch
        end
    end

end


fprintf('Reference circles manually adjusted: %d\n', ...
    sum(validAdjusted));


fprintf('\nFull Excel:\n%s\n',xlsxFile);
fprintf('\nSimple Excel:\n%s\n',simpleXlsx);
fprintf('\nQC folder:\n%s\n',qcFolder);

fprintf('============================================================\n\n');


disp(T_simple);
```

```
%% ======================================================================
% LOCAL FUNCTION
% RADIAL SYMMETRY COST
% ======================================================================

function cost = radialSymmetryCost( ...
    offsetMM, ...
    cxRef, ...
    cyRef, ...
    xData, ...
    yData, ...
    aData, ...
    bData, ...
    mmPerPixel, ...
    rMinMM, ...
    rMaxMM, ...
    binWidthMM, ...
    trimPercent, ...
    maxOffsetMM)


    dxMM = offsetMM(1);
    dyMM = offsetMM(2);


    %% ------------------------------------------------------------
    % Prevent optimizer from leaving predefined search area
    % ------------------------------------------------------------

    if hypot(dxMM,dyMM) > maxOffsetMM

        cost = ...
            1e10 + ...
            1e8*hypot(dxMM,dyMM);

        return;

    end


    %% ------------------------------------------------------------
    % Candidate center in image coordinates
    % ------------------------------------------------------------

    cx = ...
        cxRef + dxMM/mmPerPixel;


    % positive physical Y = superior
    cy = ...
        cyRef - dyMM/mmPerPixel;


    %% ------------------------------------------------------------
    % Radius from candidate treatment center
    % ------------------------------------------------------------

    rMM = ...
        hypot( ...
        xData-cx, ...
        yData-cy) ...
        * mmPerPixel;


    valid = ...
        rMM >= rMinMM & ...
        rMM <= rMaxMM;


    r = rMM(valid);

    a = aData(valid);
    b = bData(valid);


    if numel(r) < 300

        cost = Inf;
        return;

    end
```

```
%% ------------------------------------------------------------
% Radial bins
% ------------------------------------------------------------

bin = ...
      floor( ...
      (r-rMinMM) ...
      / binWidthMM) + 1;


nBins = ...
      ceil( ...
      (rMaxMM-rMinMM) ...
      / binWidthMM);


keep = ...
      bin >= 1 & ...
      bin <= nBins;


bin = bin(keep);

a = a(keep);
b = b(keep);


%% ------------------------------------------------------------
% Mean Lab color within each radial ring
% ------------------------------------------------------------

meanA = accumarray( ...
      bin, ...
      a, ...
      [nBins 1], ...
      @mean, ...
      NaN);


meanB = accumarray( ...
      bin, ...
      b, ...
      [nBins 1], ...
      @mean, ...
      NaN);


%% ------------------------------------------------------------
% Residual from perfect radial symmetry
% ------------------------------------------------------------

residual = ...
      (a-meanA(bin)).^2 + ...
      (b-meanB(bin)).^2;


%% ------------------------------------------------------------
% Robust trimmed score for each radial ring
% ------------------------------------------------------------

ringCost = nan(nBins,1);


for k = 1:nBins

      rr = residual(bin==k);

      rr = rr(isfinite(rr));


      if numel(rr) < 20
            continue;
      end


      rr = sort(rr);


      nKeep = ...
            max( ...
            1, ...
            round( ...
            numel(rr) ...
```

```
            * trimPercent/100));


        rr = rr(1:nKeep);


        ringCost(k) = ...
            median(rr);

    end


    validRings = ...
        isfinite(ringCost);


    if sum(validRings) < 8

        cost = Inf;

    else

        % Each radial ring contributes equally
        cost = ...
            median( ...
            ringCost(validRings));

    end

end
```
